# Inhibition of PDE10A-Rescued TBI-Induced Neuroinflammation and Apoptosis through the cAMP/PKA/NLRP3 Pathway

**DOI:** 10.1155/2022/3311250

**Published:** 2022-04-12

**Authors:** Jin Huang, Dang Tang, Yiqiang Cao, Yonggang Wang, Jiang Long, Lin Wei, Hai Song

**Affiliations:** ^1^Department of Neurosurgery, First Affiliated Hospital of Kunming Medical University, Kunming 650032, China; ^2^Department of Ultrasound, First Affiliated Hospital of Kunming Medical University, Kunming 650032, China

## Abstract

Phosphodiesterase 10A (PDE10A) is a dual-substrate phosphodiesterase that is highly expressed in the striatal complex. PDE10A is an important target for the treatment of ganglion dysfunction and neuroinflammation-related diseases, but its possible impact on traumatic brain injury (TBI) is still unclear. This study aims to investigate the protective effects of inhibiting PDE10A on neuroinflammation post-TBI injury and its possible molecular mechanism. The expression of PDE10A in rats and HT22 cells was determined by Western blotting. The neurological dysfunction of these rats was detected by Nissl staining, hematoxylin-eosin (HE) staining, and Morris water maze test. The activity of HT22 cells was measured by MTT. The findings of this study suggest that PDE10A is highly expressed in the brain tissue of TBI rats and HT22 cells induced by mechanical injury. Inhibition of PDE10A reduces the expression of interleukin-1*β* (IL-1*β*) and interleukin 6 (IL-6) and tumor necrosis factor alpha (TNF-*α*) in HT22 cells induced by mechanical injury to inhibit cell apoptosis. Simultaneously, inhibition of PDE10A in TBI rats reduces the time to find a visible platform in the same pool, while cAMP/PKA activator treatment alleviates all of the abovementioned phenomena. Additionally, it is further confirmed that inhibition of PDE10A activates the cAMP/PKA pathway and downregulates the expression of NRLP3. These findings demonstrate that inhibition of PDE10A exerts neuroprotection by inhibiting apoptosis and inflammation following TBI, at least partially by the cAMP/PKA/NLRP3 pathway.

## 1. Introduction

Traumatic brain injury (TBI) is the main cause of neurological dysfunction, death, and disability [1, 2]. Primary brain injury is direct physical damage to the brain tissue caused by external shocks, which is usually irreversible. Secondary brain injury includes neuroinflammation andapoptosis, which can be reversed in most cases [3, 4]. TBI leading to secondary brain injury occurs after a primary injury, subsequently contributeing to brain tissue damage and neuronal cell death [5, 6]. Brain injury causes nerve cells to activate and release pro-inflammatory factors, leading to neuroinflammation, which is the main sign of TBI. Previous studies have confirmed that brain injury induces an inflammatory response by activating neuroinflammatory mediators [7]. However, the underlying molecular mechanism of neuroinflammation post TBI is still elusive.

PDE10A is a dual-substrate phosphodiesterase that can catalyze cAMP and cGMP simultaneously. PDE10A is highly expressed in the striatal complex, which is the major entry structure to the basal ganglia [8]. Thus, PDE10A is considered to be an important target for the treatment of diseases related to ganglion dysfunction [9]. In recent years, it has been found that inhibition of PDE10A has an anti-inflammatory effect under neuroinflammatory conditions. The PDE10A inhibitor Papaverine is effective in optic neuropathy, LPS-stimulated macrophages/microglia mediated inflammation [10–12]. The PDE10A inhibitor MP-10 inhibits microglia activation in LPS-induced neuroinflammation and MPTP-induced Parkinson's disease mouse models, improving the development of neurodegenerative disease [13]. Our previous study found that PDE10A is highly expressed in TBI rats, but the specific mechanism is still unclear. Therefore, further study is needed to clarify the correlation between PDE10A and TBI.

In this study, we exposed HT22 cells to mechanical injury to mimic the neuronal inflammation caused by TBI in vitro [14, 15] and combined with TBI rat experiments to study the protective effect of inhibiting PDE10A on neuroinflammation post-TBI injury and its possible molecular mechanism.

## 2. Materials and Methods

### 2.1. Reagents and Antibodies

MP-10 was brought from Pharma Resources Inc. (Shanghai, China). The MTT assay kit was bought from Sigma-Aldrich (St. Louis, MO, USA). 8-Bromo-cAMP was brought from Selleck (Houston, Texas, USA). Antibodies PDE10A, TNF-*α*, IL-1*β*, IL-6, Bax, Bcl-2, caspase-3, NLRP3, and caspase-1 were purchased from Santa Cruz Biotechnology (Santa Cruz, CA, USA).

### 2.2. Animal Model

We followed the previous method as stated in [16]. Briefly, Sprague-Dawley rats (270–300 g, purchased from the Laboratory Animal Center of Kunming Medical University) were randomly divided into 4 experimental subgroups (*n* = 3 per group). Rats were installed in a stereotactic frame and a single metal pendulum of 1450 g was impacted on the parietooccipital bone to induce TBI. The rat in the sham group were kept under the same environmental except TBI. All protocols were conducted in accordance with guidelines set forth by the National Institutes of Health (NIH) Guide for the Care and Use of Laboratory Animals and were approved by the Ethics Committee of Kunming Medical University. Intracerebroventricular administration was performed as previously described [17]. In brief, after being anesthetized with 2% pentobarbital, rats were fixed on a stereotaxic instrument. The drugs were then injected directly into right lateral ventricles by using a 10 *μ*l Hamilton syringe (Hamilton Company, Reno, NV). We refer to Shen et al.'s coordinates: 1.5 mm below the horizontal plane of the skull and 1.0 mm and 3.2 mm horizontally [18]. MP-10 and 8-Bromo-cAMP were administered by ICV injection at 1 h before TBI modeling. The pcDNA-NLRP3 vector was injected 20 days before modeling.

### 2.3. HT22 Cell Culture and Treatments

According to the method of Rachmany et al. [14], HT22 is used to construct an in vitro sublethal stretched TBI model (mechanical injury treatment). The stretched cells maintained cell membrane integrity and function [19]. In brief, mouse hippocampal HT22 cells (BeNa Culture Collection, Beijing, China) were cultured in Dulbecco's modified Eagle's medium (DMEM) containing 10% fetal bovine serum (Gibco, Carlsbad, CA), 1% penicillin, and streptomycin (Thermo Fisher Scientific, Inc., USA). HT22 cells were inoculated in flexible membrane plates (Flexcell International Corporation, Burlington, NC). The HT22 cells were subjected to moderate stretch injury with the cell injury controller II (CIC; Custom Design & Fabrication, Richmond, VA, USA) [20]. The cell Injury Controller II applies a short-duration nitrogen pulse (50 ms) to each hole to instantaneously deform the silicone rubber membrane and achieve a predetermined degree of stretching. In the present study, the pulse injury pressure (PI) was approximately 10.8 psi.

### 2.4. HT22 Cell Viability

Cell viability was determined using the 3-(4, 5-dimethyl-2-thiazolyl)-2, 5-diphenyltetrazolium bromide (MTT) assay. Briefly, HT22 cells induced by mechanical injury were seeded in 24-well plates in DMEM containing 10% FBS, according to the manufacturer's instructions. HT-22 cells were treated with MP-10, 8-Bromo-cAMP, pcDNA-NLRP3. Next, 50 *μ*L of MTT was added to the medium for another 4 h at 37°C, After the medium was removed, DMSO was added to the plates. An automatic microplate reader was used to determine the absorbance at 470 nm. The experiment was repeated three times.

### 2.5. Western Blotting Assay

We prepared protein extracts from rats' brain tissue and HT22 cells. For rat's brain tissue, Briefly, rats were killed by decapitation; whole brains were removed and frozen at −80°C brain. Tissue lysates were prepared from frozen brain tissues, brain tissues were homogenized in RIPA buffer supplemented with protease and phosphatase inhibitors (Roche). Next, protein concentrations in the supernatant were determined using a BCA assay kit (Beyotime, Shanghai, China). An equal volume of 15 *μ*g of proteins (extracted from the rats brain tissue and HT22 cells) were separated on 5–10% SDS-PAGE gels and transferred to the polyvinylidene difluoride membrane followed by blocking in 5% skim milk, next, the following antibodies were added to detect the primary antibodies: PDE10A (1 : 1000), TNF-*α* (1 : 1000), IL-1*β* (1 : 1000), IL-6 (1 : 1000), Bax (1 : 1000), Bcl-2 (1 : 1500), caspase-3 (1 : 1000), NLRP3 (1 : 1000), caspase-1 (1 : 1500), and GAPDH (1 : 2000). Next day, the membranes were incubated with horseradish peroxidase-conjugated secondary antibodies (1 : 3000) for 1 h; the immunoblots were developed using an ECL chemiluminescence system, according to the manufacturer's instructions (Beyotime, China), and analyzed with Image J software (NIH, Bethesda, MD, USA).

### 2.6. Morris Water Maze Test

Evaluate rat's learning and memory abilities in the Morris water maze. In brief, a large circular pool was filled with water and a circular escape platform was placed in the center of the southwest. All rats underwent the place navigation task. The tracking camera and analysis software record the escape latency of the rat to the platform, and the camera record the position, swimming distance, swimming time, and swimming path of rats.

### 2.7. H&E and Nissl Staining

To analyze neuronal cell death and lesion post TBI, we follow the Hengchang et al. method [21], In brief, brain tissues of different treatments were fixed in 4% paraformaldehyde, paraffin-embedded, and cut into 4 *μ*m thick sections. The sections with different treatments were subjected to hematoxylin and eosin (H&E) staining and toluidine blue (Nissl) staining, and the tissue slides were treated in xylene solution for 3 minutes and then mounted. A light microscope was used to examine the staining images.

### 2.8. Statistical Analysis

All data were presented as mean ± SD. The data of MTT and Western blot were analyzed using *t*-test, and more than two groups was analyzed by one-way analysis of variance (ANOVA). The statistical analyses were performed using GraphPad Prism software. A value of *P* < 0.05 or *P* < 0.01 was considered as statistically significant.

## 3. Results

### 3.1. PDE10A Is Highly Expressed in TBI Tissues and HT22 Cell Lines

**Figure 1:**
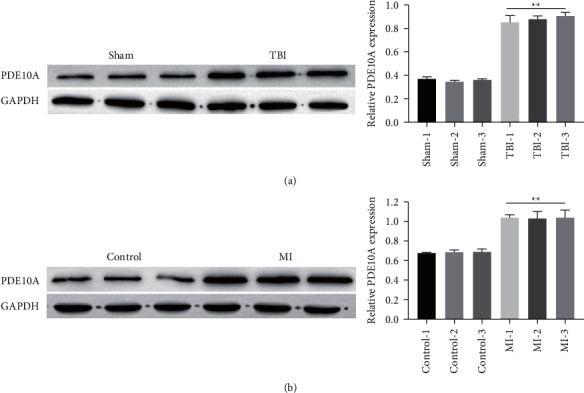
PDE10A is highly expressed in TBI tissues and HT22 cell lines. The expression of PDE10A in tissues and HT22 cells were measured by Western blotting (a, b). ^*∗∗*^Significant compared to sham/control (^*∗∗*^*P* < 0.01).

Based on previous studies [16], we detected the expression of PDE10A in TBI rats and HT22 cells. The results of Western blotting showed that the PDE10A expressed exceptionally high in TBI group compared with the sham group (Figure 1(a)). Simultaneously, we detected the expression of PDE10A in HT22 cells after 12 h mechanical injury, we found that PDE10A expression was up-regulated post mechanical injury treatment (MI; in vitro model of TBI) in HT22 cells compared to control group (Figure 1(b)).

### 3.2. PDE10A Promoted Neurological Dysfunction in TBI Rats

**Figure 2:**
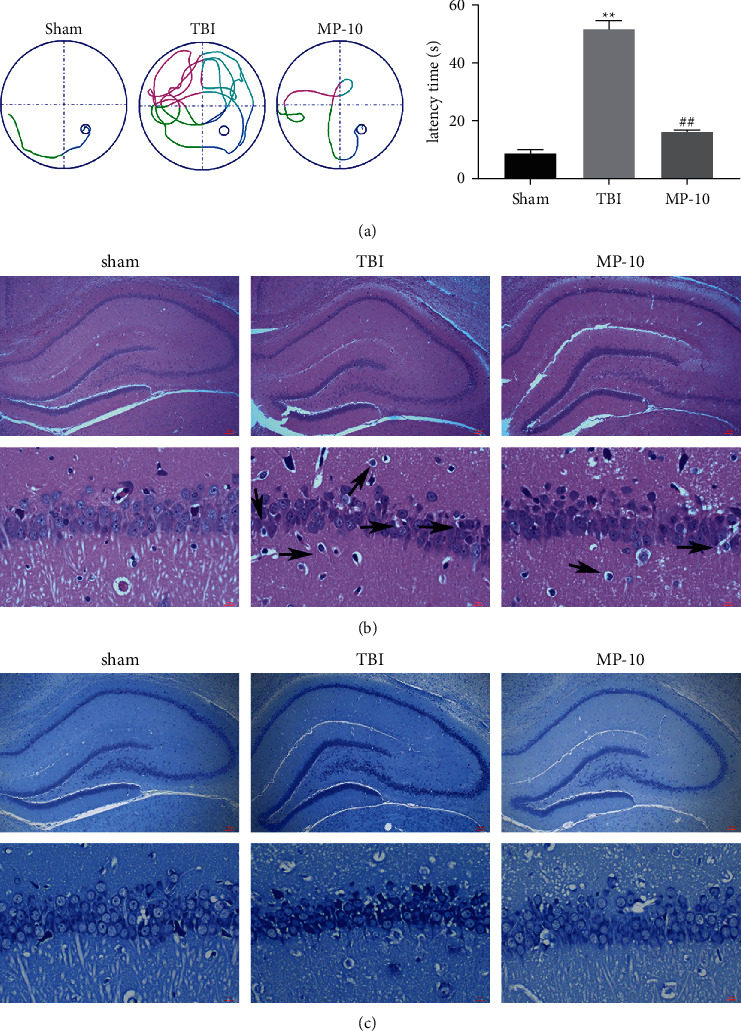
PDE10A promotes neurological dysfunction in TBI rats. Representative images of the escape track of rats in the Morris water maze test (a). The hematoxylin and eosin (H&E)-stained and Nissl-stained hippocampal sections (×100) (b) and (c). ^*∗∗*^Significant compared to sham/control (^*∗∗*^*P* < 0.01).

We further analyze the protective effect of inhibiting PDE10A on neurological dysfunction in TBI rats. Morris water maze test results showed that TBI induced a longer latency to find the platform, PDE10A inhibitor (MP-10; 3 mg/kg) shortens the time to find the platform (Figure 2(a)). Next, we use H&E staining and Nissl staining to observe the hippocampal CA1 region sections. The results of H&E staining and Nissl staining showed that the hippocampal neurons in the sham group were intact with normal morphology and obvious nucleoli. In the TBI group, hippocampal neurons are incomplete, with irregular cell contours and loose chromatin, membrane blebbing, shrunken soma, and concentrated nucleus. In addition, hippocampal neuronal density was significantly reduced. The damaged of neuronal by TBI-induced were ameliorated by prior treatment with PDE10A inhibitor (Figures 2(b) and 2(c)).

### 3.3. PDE10A Promoted Neuroinflammation of HT22 Cells Post Mechanical Injury

**Figure 3:**
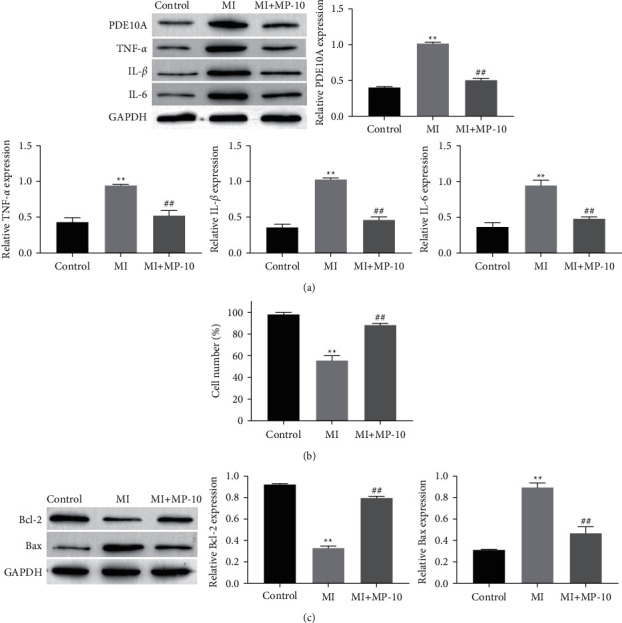
PDE10A promotes neuroinflammation of HT22 cells post mechanical injury. The expression of TNF-*α*, IL-1*β*, IL-6, Bax, Bcl-2, and caspase-3 in HT22 cells were measured by Western blotting (a) and (c). HT22 cells viability was measured by the MTT assay (b). ^*∗∗*^Significant compared to control (^*∗∗*^*P* < 0.01). ^##^Significant compared to PDE10A inhibitor treatment (^##^*P* < 0.01).

Western blotting results showed that PDE10A inhibitor provoked a loss in the expression of PDE10A compared with the control and mechanical trauma group (Figure 3(a)). In parallel, Western blotting results showed that a PDE10A inhibitor led to a significant decrease of inflammation-related proteins TNF-*α*, IL-1*β*, and IL-6 (Figure 3(a)). In addition, the results of MTT and Western blotting showed that PDE10A inhibitor improved mechanical trauma-induced apoptosis and upregulated the expression level of anti-apoptotic protein Bax (Figures 3(b) and 3(c)). Therefore, the PDE10A inhibitor alleviates neuroinflammation and apoptosis of HT22 cells post-mechanical Injury.

### 3.4. PDE10A Inhibits the cAMP/PKA Pathway

**Figure 4:**
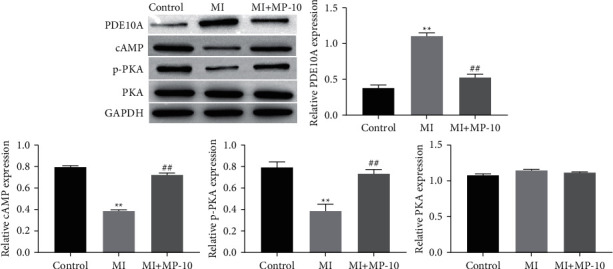
PDE10A inhibiting the cAMP/PKA pathway. The expression of cAMP p-PKA and PKA in HT22 cells were measured by Western blotting. HT22 ^*∗∗*^significant compared to control (^*∗∗*^*P* < 0.01). ^##^Significant compared to MI (^##^*P* < 0.01).

We measured the expression of cAMP/PKA pathway. Western blotting results showed that cAMP and p-PKA are significantly downregulated in mechanical trauma-induced cells. PDE10A inhibitor (MP-10; 5 *μ*M; for 1 h) activates the cAMP/PKA pathway and upregulates the expression of CAMP and p-PKA (Figure 4). Thus, the inhibition of PDE10A activates the cAMP/PKA signaling pathway.

### 3.5. cAMP/PKA Inhibits the NLPR3 Expression

**Figure 5:**
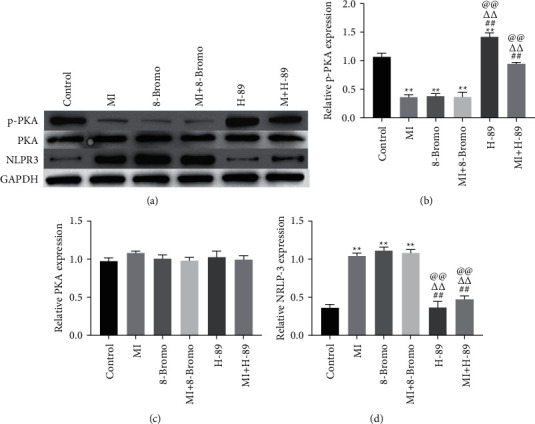
cAMP/PKA inhibits NLPR3 expression. The expression of p-PKA, PKA, and NLRP3 in HT22 cells were measured by Western blotting. HT22 ^*∗∗*^significant compared to control (^*∗∗*^*P* < 0.01). ^##^Significant compared to MI (^##^*P* < 0.01). ^ΔΔ^ was considered significant compared to cAMP/PKA activator treatment (^ΔΔ^*P* < 0.01). ^@@^Significant compared to MI + cAMP/PKA activator treatment (^@@^*P* < 0.01).

We measured the effect of cAMP/PKA on the expression of NLRP3. Western blotting results showed that cAMP/PKA (8-Bromo-cAMP; 4 *μ*M; for 1 h) activation caused the down-regulation of NLPR3. Simultaneously, the PKA inhibitor (H-89; 10 *μ*M; 1 hour) significantly upregulated the expression of NLPR3 (Figure 5).

### 3.6. cAMP/PKA Alleviated TBI Damage by Inhibiting NLPR3

**Figure 6:**
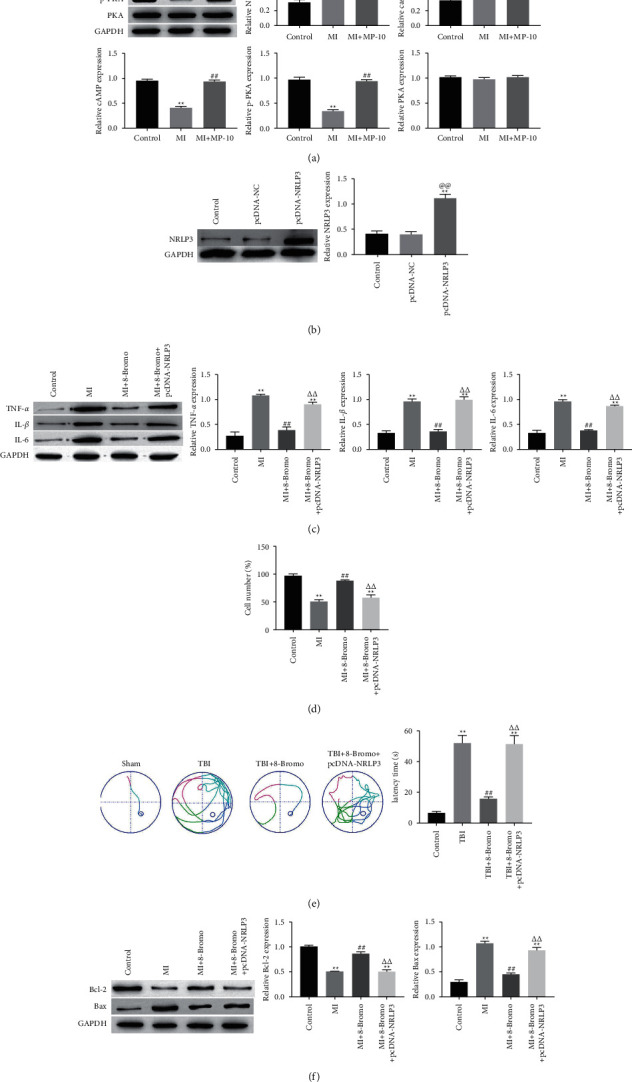
cAMP/PKA alleviated TBI damage by inhibiting NLPR3. The expression of NLPR3, caspase1, TNF-*α*, IL-1*β*, IL-6, Bax, Bcl-2, and caspase-3 in HT22 cells was measured by Western blotting (a–c) and (f). HT22 cells viability was measured by the MTT assay (d). Representative images of the escape track of rats in the Morris water maze test (e). ^*∗∗*^Significant compared to control (^*∗∗*^*P* < 0.01). ^##^Significant compared to MI/TBI (^##^*P* < 0.01). ^ΔΔ^Significant compared to cAMP/PKA activator treatment (^ΔΔ^*P* < 0.01).

We further studied the regulation of cAMP/PKA and NLRP3. Western blotting results showed that cAMP/PKA (8-Bromo-cAMP; 4 *μ*M; for 1 h) activation causes downregulation of NLPR3 and caspase-1 compared with the control and mechanical trauma group (Figure 6(a)). Simultaneously, the expression of NLRP3 was upregulated after transfection of pcDNA-NLRP3 in cells (Figure 6(b)). In addition, Western blotting results showed that cAMP/PKA activation resulted in the downregulation of inflammation-related proteins TNF-*α*, IL-1*β*, and IL-6 (Figure 6(c)). In addition, the results of MTT and Western blotting showed that cAMP/PKA activation alleviated mechanical trauma-induced apoptosis (Figures 6(d) and 6(f)). The pcDNA-NLRP3 group (50 nM transfected into HT22 cells) upregulated the expression of TNF-*α*, IL-1*β*, and IL-6 and promoted cell apoptosis compared with the cAMP/PKA activator group (Figures6(c) and 6(f)). The Morris water maze test showed that cAMP/PKA activator (8-Bromo-cAMP; 5 mg/kg) shortened the platform search time compared with the pcDNA-NLRP3 group (5 *μ*L/rat) and the TBI group (Figure 6(d)).

## 4. Discussion

Our previous study used iTRAQ-based proteomics to analyze the brain proteome of normal and different type of rat mTBI models found that the expression of PDE10A was significantly upregulated in mTBI rats [16]. Changes in PDE10A expression are expected to become a marker of disease progression, drug target identification, and treatment response in TBI. According to reports, PDE10A plays a key role in neurological complications by affecting synaptic transmission, neuronal excitability, and synaptic plasticity [13, 22]. In parallel, PDE10A has neuroinflammation- promoting effects. However, the specific mechanism of PDE10A on TBI-induced inflammation and neuronal apoptosis is still unclear. In this study, we demonstrated that inhibition of PDE10A has a protective effect on TBI-induced inflammation and neuronal apoptosis. The inhibition of PDE10A reduced the expression of TNF-*α*, IL-1*β*, and IL-6 in HT22 cells induced by mechanical injury and inhibited cell apoptosis; simultaneously, inhibiting PDE10A in TBI rats shortens the time to find a platform. In addition, PDE10A inhibited the activation of cAMP/PKA, and the inhibition of PDE10A upregulated the expression of cAMP and p-PKA. Moreover, the activation of the cAMP/PKA pathway reduced the expression of TNF-*α*, IL-1*β*, and IL-6 induced by mechanical injury through downregulation of NLRP3 and inhibiting cell apoptosis. In our study, the effects of inhibiting the anti-inflammatory and neuroprotective effects of PDE10A indicate the importance of the PDE10A/cAMP/PKA/NLRP3 pathway in mediating these effects.

TBI is accompanied by neuroinflammation [23, 24]. A rapid rise in the levels of cytokines (IL-1*β*, IL-6, and TNF-*α*) and chemokines following TBI [25] leads to the rapid development of inflammatory response [26]. In this study, we exposed HT22 cells to mechanical injury to mimic the neuronal inflammation caused by TBI in vitro. It is worth mentioning that previous studies have used LPS and mechanical injury to mimic a cellular model of induce TBI [14]. In contrast, we believe that mechanical injury induction is more in line with the damage environment caused by TBI to neurons. In HT22 cells induced by mechanical injury, IL-1*β*, IL-6, and TNF-*α* are significantly high expression, while the survival rate of HT22 cells is reduced. Recently, PDE10A has been increasingly appreciated as important mediators of neurological dysfunction progression. PDE10A can regulate synaptic transmission, neuronal excitability, and synaptic plasticity, playing key roles in neurological dysfunction [27]. In addition, PDE10A abnormal expression in neurological and psychiatric disorders [8, 28]. In order to understand the expression of PDE10A after TBI, we determined the amount of PDE10A protein in vivo and in vitro after TBI injury. We found that PDE10A is highly expressed in TBI rats. Simultaneously, when we inhibited the expression of PDE10A in rats, it effectively shortened the time for rats to find the platform and alleviated brain damage in rats. On the other hand, the inhibition of PDE10A significantly downregulated the expression of TNF-*α*, IL-1*β*, and IL-6 in mechanical trauma-induced HT22 cells and inhibited apoptosis. Therefore, the increase of PDE10A expression is correlated with TBI pathogenesis.

cAMP is the second messenger, which plays a major role in cytokine secretion and cell signal transduction [29, 30]. In cells, the expression of cAMP is regulated by adenosine A2A receptor (A2AR) and PDE10A [31, 32]. The inhibition of PDE10A leads to increased intracellular cAMP levels and activates the PKA signaling pathway [29]. PKA-phosphorylation promotes CREB transcriptional activation and further promotes the combination of CREB and transcriptional co-activator CBP to form a complex that blocks the transcription of inflammatory genes [33]. In this study, cAMP/PKA is low expressed in HT22 cells induced by mechanical injury, and it was found that activation of cAMP/PKA inhibits the inflammatory response of TBI. In addition, PDE10A is overexpressed in TBI rats and HT22 cells induced by mechanical injury, demonstrating a correlation between the PDE10A/cAMP/PKA levels and TBI.

The cAMP/PKA pathway contributes to the resolution of inflammation [34, 35]. This anti-inflammatory effect is closely related to PKA-phosphorylation-mediated upregulation of the anti-inflammatory cytokine and inhibition of proinflammatory cytokines such as IL-1*β*, IL-6, and TNF-*α* [10, 36]. In addition, studies have shown a prominent role of the cAMP/PKA signaling pathway in regulating NLRP3-related inflammation [37]. As a core part of the inflammatory response, activation of the NLRP3 inflammasome promoted the release of proinflammatory cytokines [38, 39]. Simultaneously, cAMP has been shown to inhibit the phosphorylation of NLRP3 [40]. In our studies, we found that pcDNA-NLRP3 upregulated the expression of TNF-*α*, IL-1*β*, and IL-6 and promoted cell apoptosis in HT22 cells induced by mechanical injury compared with the cAMP/PKA activator group.

In conclusion, our results indicate that PDE10A is highly expressed post-TBI. inhibition of PDE10A exhibits a neuroprotective effect against TBI by relieving neuroinflammation via downregulates the NLRP3 inflammasome through activation of the cAMP/PKA pathway.

## Data Availability

The data used to support the findings of this study are available from the corresponding author upon reasonable request.
